# Cholate Conjugated Polymeric Amphiphiles as Efficient Artificial Ionophores

**DOI:** 10.1021/acsapm.0c01182

**Published:** 2021-01-04

**Authors:** Subhasish Sahoo, Jawad ur Rehman, Muhammad Raza Shah, Priyadarsi De, Paolo Tecilla

**Affiliations:** † Polymer Research Centre and Centre for Advanced Functional Materials, Department of Chemical Sciences, Indian Institute of Science Education and Research Kolkata, Mohanpur, Nadia, West Bengal 741246, India; ‡ H. E. J. Research Institute of Chemistry, International Center for Chemical and Biological Sciences, University of Karachi, Karachi, Sindh 75270, Pakistan; § Department of Chemical and Pharmaceutical Sciences, University of Trieste, via Giorgieri 1, I-34127 Trieste, Italy

**Keywords:** amphiphilic copolymers, cholic acid, artificial ionophore, cellular membrane, ion transport

## Abstract

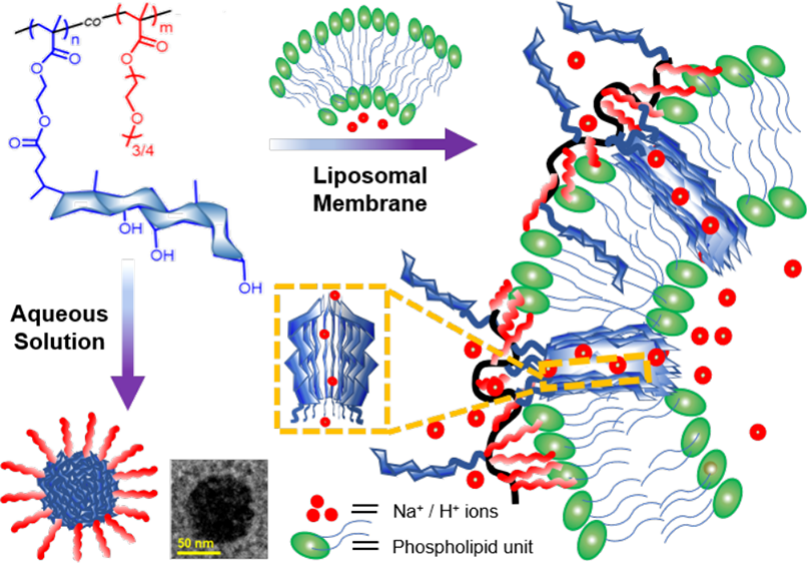

A family of amphiphilic copolymers containing hydrophobic cholate pendants has been prepared by copolymerization of cholic acid-based monomer 2-(methacryloxy)-ethyl cholate (MAECA) with polyethylene glycol methyl ether methacrylate (PEGMA). The polymers differ for the content of MAECA that increases from 0 to 35%. The copolymers partition within liposomes and display potent ionophoric activity forming large pores in the membrane and allowing the leakage of small inorganic ions (H^+^, Na^+^) and of large polar organic molecules (calcein). Their activity is strictly correlated to the content of cholic acid subunits, increasing as the fraction of cholate moiety increases.

The current decade evidences an increasing interest in artificial ionophores^1,2^ because of their potential appliances in various technological^3^ and biomedical fields.^4^ These ionophores operate via transportation of inorganic and molecular ions across the semipermeable cellular membrane. Nature has developed several ionophores for proper functioning of fundamental biological processes like muscle contraction, signal transduction, nerve impulse, hormonal regulation, apoptosis, etc.^5^ The dysfunctional alteration of membrane permeability causes restriction over ionic transport, which leads to Parkinson’s disease, Alzheimer’s disease, cystic fibrosis, and many other diseases.^6^

Several artificial ionophores were reported on the basis of polypeptides,^7^ DNA-based complexes,^8^ nonporous materials,^9^ and amphiphilic synthetic scaffolds.^10^ In this context, polyhydroxylated steroidal amphiphiles are highly convenient for mimicking the natural protein-based ion channels. They can mimic the “barrel stave” conformation of naturally occurring ionophore, i.e., antifungal macrolide amphotericin B (AmB), to span the membrane bilayer. Among all hydroxylated steroids, cholic acid derivatives are found to be useful as highly efficient artificial ionophores^11^ due to their peculiar facial amphiphilicity.^12^ De Riccardis et al. reported the implication of cholic acid on the ionophore purpose.^13,14^ Regen and co-workers showed the effectiveness of tetrameric cholic acid in the ion transport application.^15^ A few more examples of ionophores based on mono or oligomeric cholic acid derivatives were reported in the literature.^16,17^

To take advantage of polyvalent interactions (multivalency)^18^ by synthetic polymers, we became interested in the incorporation of cholate moieties into the side chain of the polymeric backbone and in the study of their ionophoric behavior. The synthetic procedure to access cholic acid-based polymers with a suitable aqueous solubility and desired molecular weight was reported in 2014 by De and co-workers.^19^ Herein, we copolymerized cholic acid-based monomer 2-(methacryloxy)-ethyl cholate (MAECA) with polyethylene glycol methyl ether methacrylate (PEGMA) to synthesize polymers with amphiphilic behavior (Scheme 1a) via a reversible addition–fragmentation chain transfer (RAFT) process. RAFT polymerization allows the control of the molecular weight of the polymers by changing [monomer]/[CTP] ratio (where CTP is 4-cyano-4-(thiobenzylthio)pentanoic acid; see the Supporting Information), and the content of MAECA in the polymer was varied to incorporate different amounts of cholate moiety into the copolymer side chain (Table S1). The integration of PEGMA gave aqueous solubility to the copolymers. Since the two monomers have different reactivity ratios,^19^ we got the homopolymer of PEGMA (PPEGMA) and copolymers (SCP1, SCP2, and SCP3 in Table S1) with MAECA incorporation close to the feed composition. Polymer molecular weight and monomer ratio were chosen to have polymers with a roughly similar number of monomers and increasing hydrophobic/hydrophilic balance.

**Scheme 1:**
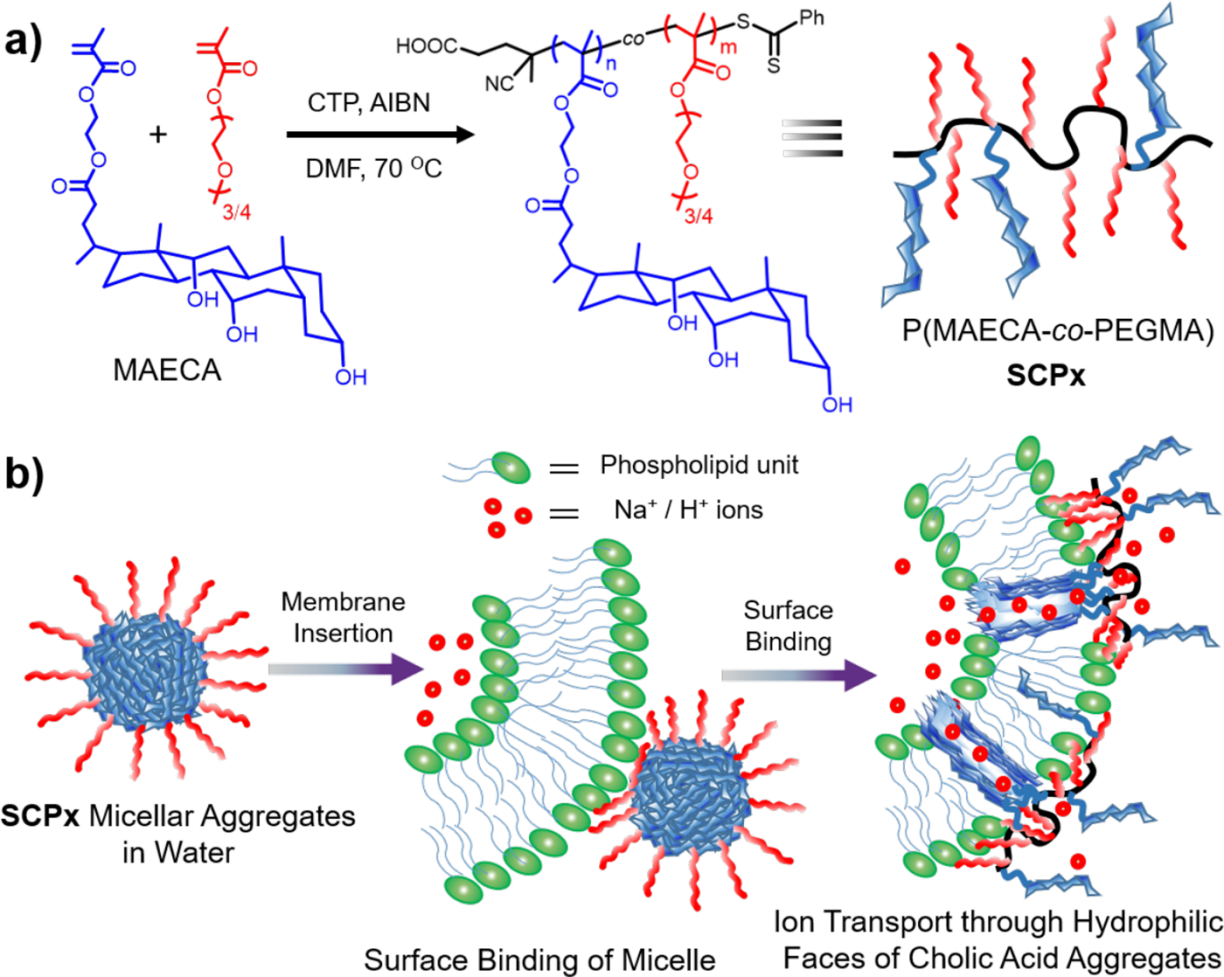
(a) Synthetic Strategy of Cholic Acid-Based Amphiphilic Copolymers and (b) Schematic Illustration of Their Ion Transportation Performance

All the polymers were nicely soluble in aqueous medium. Polymers were characterized by ^1^H NMR spectroscopy (Figure S1) and size exclusion chromatography (SEC) (Table S1). Since the amphiphilic copolymers contain hydrophobic cholate pendants and hydrophilic PEGMA moieties, they self-assembled in aqueous medium as confirmed by dynamic light scattering (DLS) (Figure S2), ^1^H NMR data (Figure S3), a critical aggregation concentration (CAC) study by fluorescence spectroscopy (Figure S4), and transmission electron microscopy (Figure S5).

The ionophoric activity of the polymers was preliminarily assessed using the 8-hydroxypyrene-1,3,6-trisulfonic acid trisodium salt (HPTS) assay (Figure 1a).^20^ HPTS is a water-soluble fluorescent pH indicator with a p*K*_a_ of 7.2. In this experiment, the dye is trapped in the inner water pool of large unilamellar liposomes (100 nm diameter) made by egg yolk phosphatidylcholine (EYPC). The lipid suspension is prepared in a 100 mM NaCl water solution buffered at pH 7 and, after the addition of the ionophore, a pH gradient is established across the phospholipid membrane by the external addition of NaOH. The increase of the HPTS fluorescence emission in response to the applied transmembrane pH-gradient indicates basification of the inner water pool, which may derive from either H^+^ efflux or OH^–^ influx, counterbalanced by the opposite transport of ions of the same charge (Na^+^ or Cl^–^) or by the symport of ions of the opposite charge (Cl^–^ or Na^+^). Then, after 350 s, a solution of Triton X-100 is added to lyse the liposome, allowing one to record the maximum fluorescence intensity change, which is used to normalized the fluorescence emission data (see the Supporting Information for details; Figure S6). Therefore, the increase in the fluorescence emission of HPTS is directly related to the ability of the polymer to promote the pH gradient discharge by facilitating the movement of ions across the membrane.

**Figure 1:**
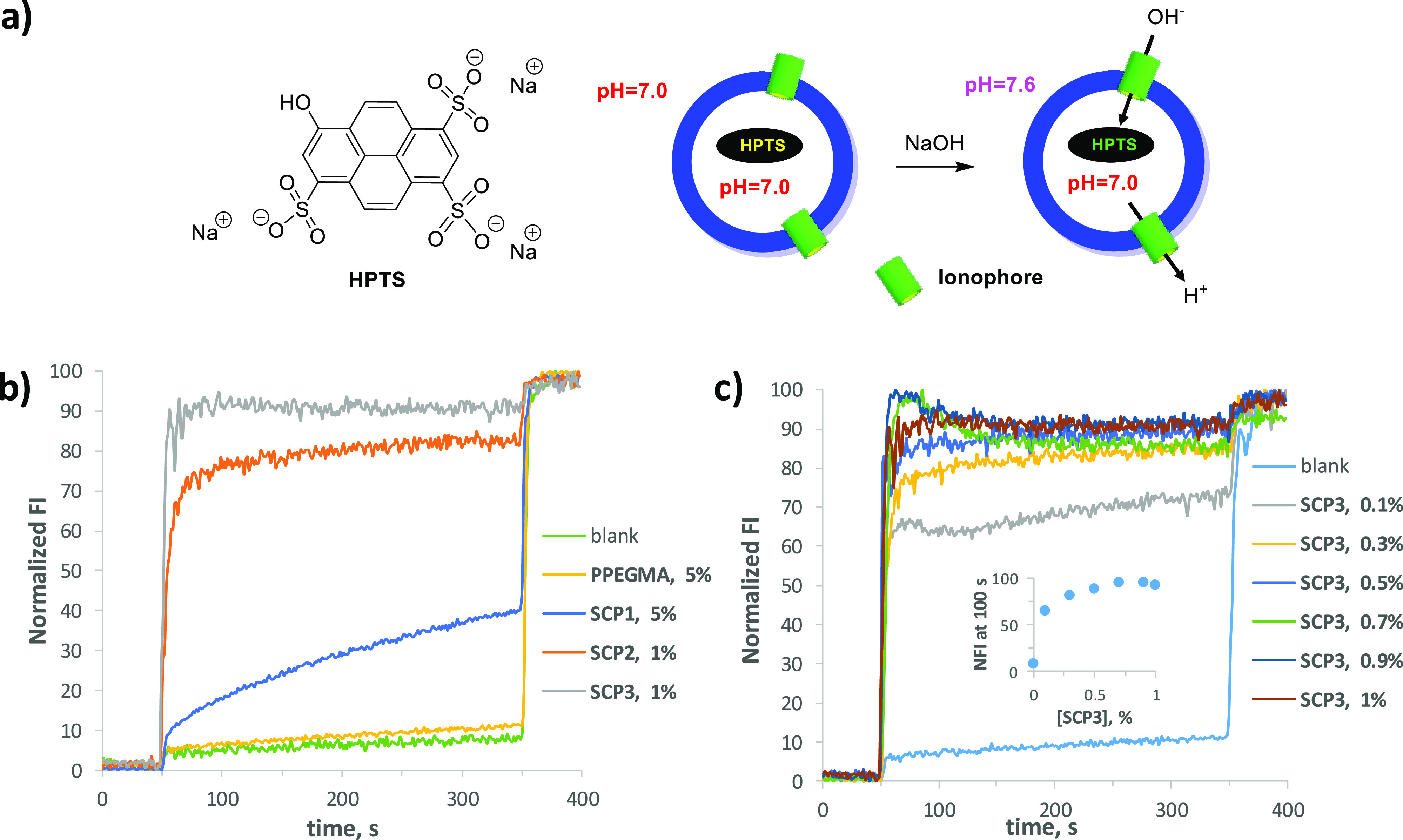
(a) Structure of HPTS (left) and schematic illustration of the HPTS assay (right) in which the polymer forms pores promoting the transport of H^+^ and/or OH^–^. (b) Normalized fluorescence time course of HPTS fluorescence emission (FI) after the addition of the base (time 50 s, 50 μL of 0.5 M NaOH) to EYPC large unilamillar vesicles (LUVs) (100 nm diameter) loaded with HPTS (0.1 mM HPTS, 0.17 mM lipid concentration, 25 mM HEPES, 100 mM NaCl, pH 7.0; total volume: 3 mL) in the presence of the different polymers. (c) Normalized fluorescence change in HPTS fluorescence emission (FI) as a function of time in the presence of different concentrations of SCP3. Inset: FI measured at 100 s as a function of SCP3 concentration. The concentrations of polymers are reported in the figures and are given in mol % with respect to the concentration of lipids.

Figure 1b shows the kinetic profiles obtained with the different polymers in the HPTS assay. The ionophoric activity increases with the increase of the MAECA content in the polymer. Indeed, PPEGMA is almost inactive, and its kinetic trace is almost superimposable to the control experiment recorded in the absence of ionophore. On the other hand, the activity increases strongly on going from SCP1 to SCP3, also taking in account that the kinetic profiles in Figure 1b are recorded at a concentration of SCP1 five times higher with respect to the concentration of SCP2 and SCP3. For these two more active compounds, the dependence of the ionophoric activity from the concentration of polymer was recorded. With an increase in the concentration of SCP3, the activity increases and the collapse of the pH gradient is very fast, almost immediate, as signaled by the fluorescence intensity jump that occurs in the first few seconds after the base pulse (Figure 1c). This initial jump accounts for almost the total pH jump registered, and its magnitude increases with the concentration of polymer as shown in the inset of Figure 1c in which the normalized fluorescence intensity recorded at 100 s is reported as a function of the polymer concentration. For example, at the lowest concentration of polymer investigated (0.1%) and after 100 s, the normalized fluorescence intensity is about 60 and remains almost constant up to the addition of the detergent. On the other hand, at higher polymer concentration, the pH gradient is fully collapsed in the first few seconds of kinetics. The same behavior, although shifted toward higher polymer concentrations, is observed with SCP2 (Figure S7). This very fast and concentration dependent collapse of the pH gradient is suggestive of the formation of large pores, which transport cations and/or anions at a high rate.^21^ An increase in the concentration of polymer increases the fraction of liposomes containing at least one pore forming molecule, and when the liposome population is saturated with polymer, the pH gradient is fully collapsed very rapidly. The efficiency of the process increases with the fraction of MAECA in the polymer, indicating that the cholate moieties are responsible for the observed ionophoric activity. This is likely the effect of two combined factors: (i) the higher hydrophobicity of the MAECA with respect to PEGMA drives the partition equilibrium of the polymers toward the membrane; (ii) the cholic acid structure self-assembles in the membrane forming the pore, and the more MAECA present, the more effective is the process.

The very fast pH discharge process observed in the HPTS assay is indicative of the formation of large pores in the membrane but does not allow a fine characterization of the kinetic processes. We therefore switched to the calcein leakage assay, which is more suitable for the study of large pore forming molecules (Figure 2a).^22^ Calcein is a water-soluble self-quenching fluorescent dye, which is not membrane permeable. In this experiment, calcein is trapped at a high concentration (50 mM) in the inner water pool of large EYPC unilamellar liposomes (100 nm diameter). In these conditions, due to the high concentration of the dye, its fluorescent emission is almost totally self-quenched. The formation of transmembrane pores promotes the leakage of the dye from the inner water pool of the liposomes and its dilution in the bulk water. As a consequence, the self-quenching process is no more effective and the emission intensity of the dye strongly increases. Therefore, an increase of calcein fluorescence emission is directly related to the formation of transmembrane pores large enough to allow the transport of the dye. Figure 2b shows the effect of the different polymers in the calcein leakage from the EYPC liposomes. In the experiment, the polymer (1% concentration) is added at 50 s and the liposomes are lysed with Triton X-100 at 450 s (Figure S8). The most active polymer is SCP3 followed by SCP2 and SCP1, while PPEGMA is nonactive and its kinetic traces are completely overlapped to that of the blank experiment. Again, the ability of the polymers to form transmembrane pores follows the increases in their content of cholic acid moiety, and this is clearly illustrated by the histogram in Figure 2c that shows the normalized amount of calcein released at 400 s in the presence of a 1% concentration of the different polymers.

**Figure 2:**
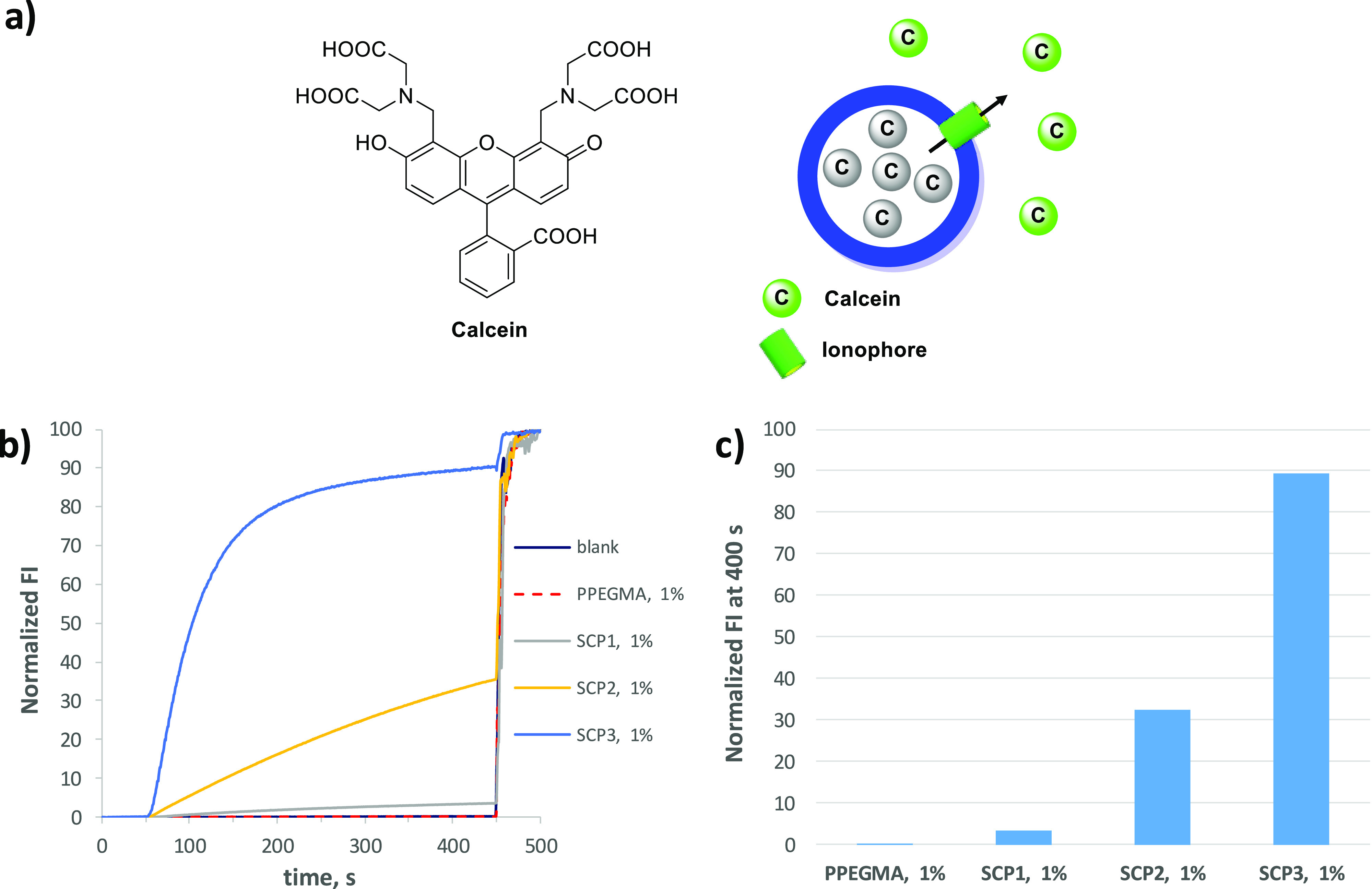
(a) Structure of calcein and schematic illustration of the leakage assay. At a high concentration, the calcein emission is self-quenched (gray color) while, upon leakage and dilution in the bulk buffer, the emission intensity strongly increases (green color). (b) Time course of calcein leakage from EYPC LUVs (100 nm diameter, 50 mM calcein, 1 mM HEPES, pH 7.4, 0.1 mM lipids concentration) in the presence of the different polymers (1% concentration). The polymer was added at 50 s, and the liposomes were lysed with Triton X-100 (40 μL, 5% water solution) at 450 s. (c) Percentage of calcein leakage after 400 s upon addition of 1% of the different polymers. The concentrations of polymers are given in mol % with respect to the concentration of lipids.

With the exception of PPEGMA, which is inactive even at high concentrations, the leakage activity of SCP1–3 increases with their concentration (Figures S9 and S10), although to a different extent. Figure 3 displays the activity concentration profiles for SCP3 and SCP2 obtained by plotting the normalized FI measured at 400 s as a function of the concentration of added polymer. In the case of SCP3, the activity increases rapidly with the concentration of polymer reaching a plateau, accounting for ca. 90% of calcein release, above the 0.5% polymer concentration. Thus, the leakage process is practically complete in 400 s in the presence of only 0.5% polymer, which corresponds to a 9.35 μg/mL concentration. SCP2 is less active, and after 400 s, only 40% of calcein is released in the presence of the concentrations of polymer between 1.5% and 2%. Above this concentration, the percent of leakage decreases when the concentration of the polymer is increased. This effect is probably related to a competition between homoaggregates of the polymer formed in the bulk water and the liposomes that limits the partition of the polymer in the membrane. An increase in the concentration of the polymer shifts the equilibrium toward the homoaggregates, thus decreasing the fraction of polymer partitioned in the membrane and affecting the overall leakage activity observed. This effect is not evident in the HPTS assay (Figure S7b), and this is likely related to the different experimental conditions of the two experiments, which may affect the position of the partition equilibrium. SCP1 is the less active polymer, and only about 10% of calcein is released after 400 s in the presence of a 5% concentration of the polymer (Figure S9).

**Figure 3:**
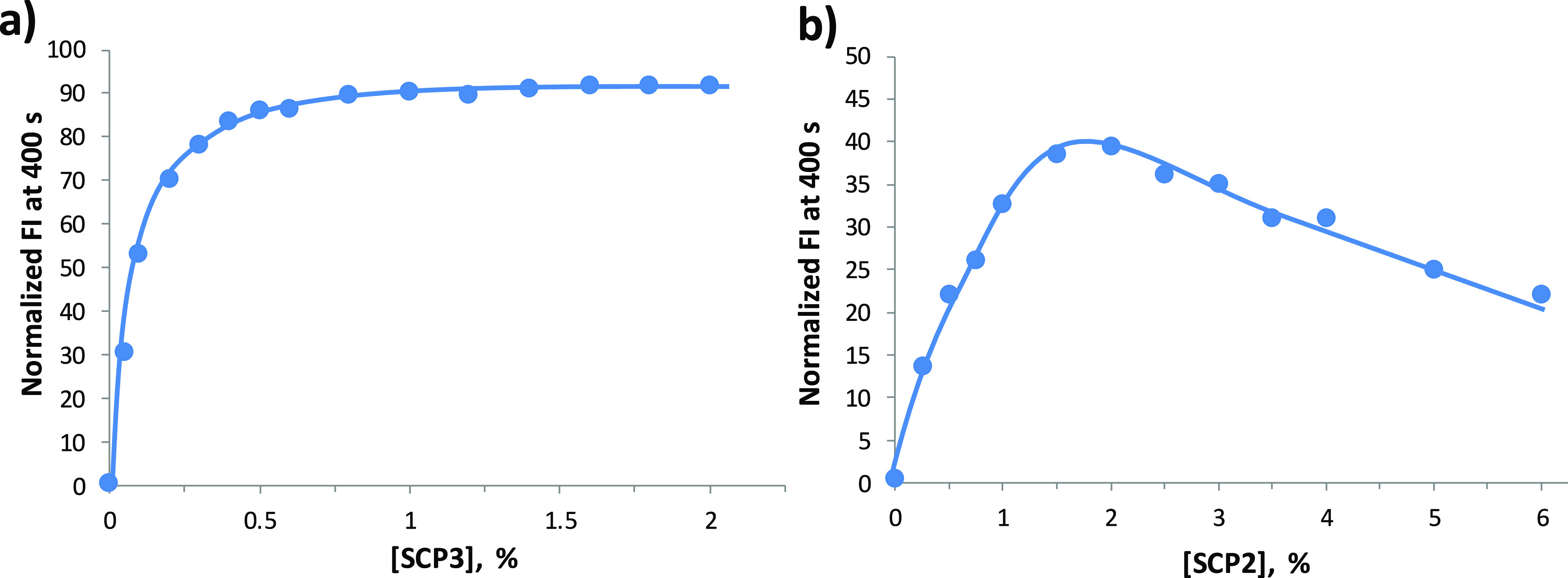
Percent leakage of calcein after 400 s as a function of SCP3 (a) and SCP2 (b) concentration. The concentrations of polymers are given in mol % with respect to the concentration of lipids (0.1 mM). The original kinetic profiles are reported in Figure S10.

Overall, the data on the ionophoric activity collected for the different polymers depicts the mechanistic scheme illustrated in Scheme 1b. PPEGMA is too hydrophilic, and it does not interact with the liposomes. However, with an increase in the content of MAECA, the lipophilicity of the polymers increases and they form aggregates in water, exposing the hydrophilic polyethylene glycol chains and burying the more lipophilic cholic acid moieties in the interiors of the aggregate to engage the stabilization of hydrophobic interactions. In the presence of liposomes, an equilibrium is established between the homoaggregate and the lipophilic phospholipid membrane. The polymers, depending on their lipophilicity, tend to partition in the membrane, likely inserting at least a fraction of the cholic acid appendages in the phospholipid bilayer and exposing the hydrophilic portions to the bulk water. Due to their facial amphiphilicity, the cholic acids self-assemble in the membrane forming pores with the hydrophobic steroid skeleton in contact with the phospholipid hydrocarbon chains and with the polar hydroxyl groups lining the interior of the pore.^13^ These pores are rather large and probably little structured and allow the transport of small ions as well as of the large calcein dye. The ionophoric ability of the polymers is therefore directly related to the content of MAECA because as the fraction of MAECA increases the lipophilicity of the polymer increases, driving the partition equilibrium toward the liposome, and the number of ionophorically active subunits increases as well. As a consequence, the highest activity is observed with SCP3 and decreases on going from SCP3 to SCP1.

In conclusion, amphipathic polymers can be easily obtained by RAFT polymerization of a cholic acid-based monomer with a polyethylene glycol pendant monomer. These polymers form large pores in a phospholipid membrane, promoting the leakage of ions and of large polar molecules, and their activity is strictly correlated to the content of cholic acid subunits, increasing as the fraction of cholic acid increases. Further studies are underway to broaden the scope of these findings and to investigate the biological activity of these new polymeric synthetic ionophores.

## Supporting Information Available

Experimental details, various characterization data, HPTS and calcein assay protocols, and ionophoric activity data (PDF)
