# Supplementary material for: Cholate Conjugated Polymeric Amphiphiles as Efficient Artificial Ionophores
Source: ACS Appl Polym Mater. 2021 Jan 4;3(2):588–93. doi: 10.1021/acsapm.0c01182 (PMC8025732; doi:10.1021/acsapm.0c01182)
Supplement: Supplementary file 1 — ap0c01182_si_001.pdf [file ap0c01182_si_001.pdf]

Supporting Information for

Cholate Conjugated Polymeric Amphiphiles as Efficient Artificial Ionophores

Subhasish Sahoo,[a,#] Jawad ur Rehman,[b,c,#] Muhammad Raza Shah,[b] Priyadarsi De,[a,*] Paolo Tecilla[c,*]

[a]Polymer Research Centre and Centre for Advanced Functional Materials, Department of Chemical Sciences, Indian Institute of Science Education and Research Kolkata, Mohanpur - 741246, Nadia, West Bengal, India.

[b]H. E. J. Research Institute of Chemistry, International Center for Chemical and Biological Sciences, University of Karachi, Karachi - 75270, Pakistan.

[c]Department of Chemical and Pharmaceutical Sciences, University of Trieste, via Giorgieri 1, I-34127, Trieste, Italy.

# These authors contributed equally to this work.

* Corresponding Authors: E-mails: p_de@iiserkol.ac.in (PD); ptecilla@units.it (PT)

Experimental section

Materials. 2-Hydroxyethyl methacrylate (HEMA, 97%), dicyclohexylcarbodimide (DCC, 99%), 4-dimethylamino pyridine (DMAP, 99%), anhydrous *N*,*N*-dimethylformamide (DMF, 99.9%) and cholic acid (CA, $\geq$ 98%) were purchased from Sigma and used without any further purification. Polyethylene glycol methyl ether methacrylate (PEGMA, average molecular weight = 300 g/mol) was purchased from Sigma (99%) and purified prior to polymerization by passing through a basic alumina column. 2,2'-Azobisisobutyronitrile (AIBN, Sigma, 98%) was

recrystallized from methanol. 4-Cyano-4-(thiobenzylthio)pentatonic acid (CTP) was synthesized following the standard literature procedure.[1] The cholic acid conjugated vinyl monomer 2-(methacryloxy)-ethyl cholate (MAECA) was synthesized by the coupling reaction of cholic acid with HEMA in the presence of DCC and DMAP as reported previously.[2] NMR solvents such as chloroform-*d* ($CDCl_3$, 99.8% D), D_2O (99.9% D) and dimethyl sulphoxide-d_6 (DMSO-d_6, 99.8% D) were obtained from Cambridge Isotope Laboratories, Inc., USA. The solvents like hexanes (mixture of isomers), tetrahydrofuran (THF) and dichloromethane (DCM) were purified by following general literature procedure.

Instrumentation. The molecular weights and molecular weight distributions (dispersity, *Đ*) values of the as-synthesized polymers were determined by size exclusion chromatography (SEC) using poly(methyl methacrylate) (PMMA) calibration in DMF at 40 ^{o}C. The instrument contains Waters Model 1515 HPLC pump, Waters 2414 refractive index (RI) detector, one PolarGel-M guard column (50 × 7.5 mm) and two PolarGel-M analytical columns (300 × 7.5 mm). The ^{1}H NMR spectra were acquired in a Bruker Avance^{III} spectrometer operating at 500 MHz. Dynamic light scattering (DLS) study was performed using a Malvern Zetasizer Nano ZS instrument (Malvern, UK) equipped with an avalanche photodiode detector with high quantum efficiency, a 4 mV He−Ne laser operating at λ = 633 nm (scattering angle = 173°), and an ALV/LSE-5003 multiple tau digital correlator electronics system. Polymer solutions (1.0 mg/mL) were filtered through 0.45 μm syringe filter prior to DLS measurements. Fluorescence measurements were conducted using Horiba JobinYvon (Fluoromax-3, Xe-150 W, 250-290 nm) spectrophotometer. Morphology of the copolymeric nanoparticles were evaluated by transmission electron microscopy (TEM) using a JEOL JEM-2100F instrument at 200 kV. TEM

samples were prepared by drop-casting an aqueous solution of respective polymers (0.1 mg/mL) on a carbon-coated copper grid and then dried under vacuum for few hours at room temperature.

Synthesis of statistical copolymers. A representative copolymerization procedure was as follows: MAECA (0.190 g, 0.365 mmol), PEGMA (0.110 g, 0.365 mmol), CTP (3.40 mg, 12.2 μmol), AIBN (0.40 mg, 2.43 μmol; 0.08 g solution of 10.4 mg AIBN in 2.09 g DMF) and DMF (1.2 g) were taken in a 20 mL septa sealed glass vial equipped with a magnetic stir bar. The vial was purged with dry N_2 for 15 min and placed in a preheated reaction block at 70 ^{o}C. After 5 h, the polymerization reaction was stopped by cooling the vial in an ice-water bath and exposed to air. Finally, the copolymer was purified by repeated precipitation with hexanes from a THF solution to remove DMF, unreacted monomers and CTP from the polymer matrix and dried under high vacuum at 40 ^{o}C obtaining reddish solid polymer. The feed ratios of monomers were varied to yield copolymers of various compositions and we named those copolymers as **SCP1**, **SCP2** and **SCP3** (Table S1). By a similar method, we also carried out homopolymerization of PEGMA to prepare the corresponding homopolymer, **PPEGMA**.

Table S1. Experimental results obtained from the RAFT homopolymerization of PEGMA and copolymerization of PEGMA with MAECA.

Polymer[a]	% MAECA content in feed	Conv.[b] (%)	% MAECA content in polymers[c]	$M_{n,theo}$[d] (g/mol)	$M_{n,SEC}$[e] (g/mol)	$Đ$[e]	$M_{n,NMR}$[c] (g/mol)
**PPEGMA**	00	59	00	11000	16200	1.09	10500
**SCP1**	10	67	13	13300	18000	1.08	14300
**SCP2**	20	74	28	15600	21000	1.07	18400
**SCP3**	30	82	35	18300	25000	1.08	18700

[a][Monomer]:[CTP]:[AIBN] = 60:1:0.2 in DMF at 70 ^{o}C, polymerization time = 5 h. [b]Determined by gravimetric analysis based on the amount of total monomer feed. [c]Calculated from ^{1}H NMR study. [d]$M_{n,theo}$ = {([Monomer]/[CTP] × average molecula weight (*MW*) of monomers × Conv.) + (*MW* of CTP)}. [e]Obtained by SEC analysis in DMF eluent.

Characterization of polymers. CTP is an efficient chain transfer agent for the RAFT polymerizations of both MAECA and PEGMA in DMF at 70 ^{o}C, and produced polymers with controlled molecular weights and narrow $Đ$ values.[1,2] Thus, statistical copolymerization of MAECA with PEGMA were carried out *via* RAFT method using CTP as RAFT agent at [monomer (M)]/[CTP]/[AIBN] = 60:1:0.2 (Scheme 1). After purification, copolymers were characterized by SEC and ^{1}H NMR spectroscopy and results are summarized in Table S1. The SEC RI traces for all the three copolymers were symmetric and unimodal in nature. From the SEC study, number average molecular weight ($M_{n,SEC}$) and $Đ$ values were obtained for the copolymers and results are shown in Table S1. Theoretical number average molecular weight, $M_{n,theo}$ = {([M]/[CTP] × average molecular weight (*MW*) of monomers × Conv.) + (*MW* of CTP)} was predicted from stoichiometry and monomer conversion for all the polymers (Table S1). Table S1 shows somewhat higher $M_{n,SEC}$ values for the polymers compared to the corresponding $M_{n,theo}$ values, although narrow $Đ$ values for all the copolymers are obtained. This discrepancy could be due to the use of conventional calibration curve while determining the $M_{n,SEC}$ values from SEC analysis.

The percentage of co-monomer units in the copolymer chain was determined from their ^{1}H NMR spectra recorded in DMSO-d_6. Peak positions are assigned in Figure S1. The compositions of copolymers (Table S1) were determined from the integration ratio of intensities of –OCH_3 protons at 3.25 ppm from PEGMA units to the –C(O)-O-CH_2-CH_2-O-C(O)- methylene protons at 3.93-4.34 ppm from the MAECA fragment (after subtracting the peak area contributed by the PEGMA units in the region). The $M_{n,NMR}$ values of P(MAECA-*co*-PEGMA) copolymers were calculated by comparing the integration values of chain end phenyl protons at 7.44-7.84 ppm from CTP moiety to the characteristic protons at 3.25 ppm (-OCH_3) from PEGMA units and –

C(O)-O-CH_2-CH_2-O-C(O)- protons at 3.93-4.34 ppm from MAECA units. For these copolymers, $M_{n,NMR}$ = [(DP_{MAECA} × MW_{MAECA}) + (DP_{PEGMA} × MW_{PEGMA}) + molecular weight of CTP], where *DP* and *MW* are the degree of polymerization and molecular weight of the monomer, respectively.[2] The $M_{n,NMR}$ values of all the polymers are listed in Table S1, which matches nicely with the $M_{n,theo}$ values.

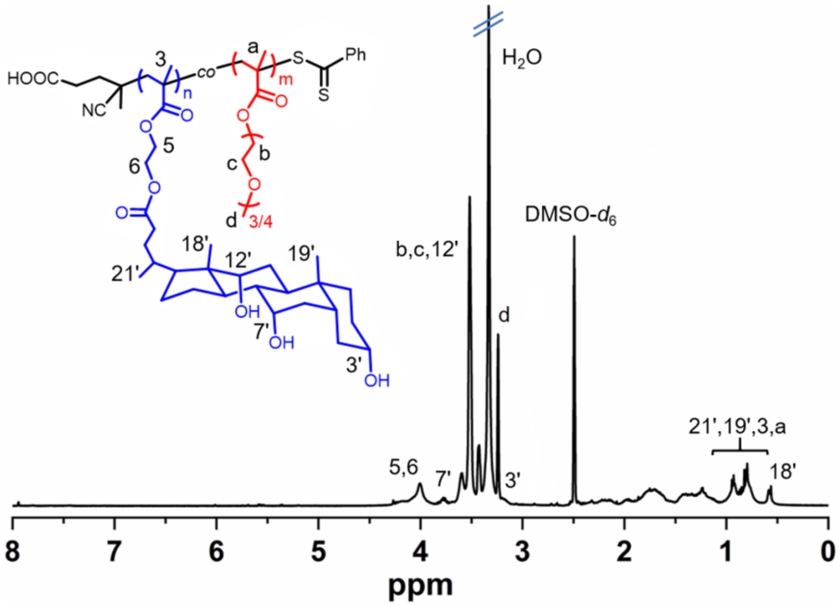

Figure S1. ^{1}H NMR spectrum of **SCP3** copolymer in DMSO-d_6 solvent.

DLS measurements. To access the hydrated state self-assembly behaviour of the synthesized cholate containing polymers, we performed DLS study both in water and DMSO (Figure S2). The polymers in DMSO showed smaller hydrodynamic diameters (D_h) which increased in aqueous media. For **SCP1** the D_h value in DMSO was 4.1 ± 2.0 nm which increases to 92 ± 4.0 nm in water. Again, for **SCP2** the D_h value increases from 4.5 ± 3.0 nm to 94 ± 4.0 nm for DMSO to water as solvent. Whereas the D_h enhances from 5.2 ± 2.0 nm in DMSO to 95 ± 3.0 nm in water for **SCP3** polymer. The cholic acid based polymers remain as unimeric chains in organic solvent like DMSO (good solvent for both the comonomers), whereas they undergo

aggregation when placed in aqueous environment through hydrophobic interactions caused by the cholic acid moiety present in polymeric entity (note that the MAECA moiety is not soluble in water, whereas PEGMA unit is nicely soluble in water). Thus, the hydrophilic PEG chains enjoys water-periphery in aqueous phase DLS experimental condition. Data of this investigational outcome are comparable with our earlier report.[2]

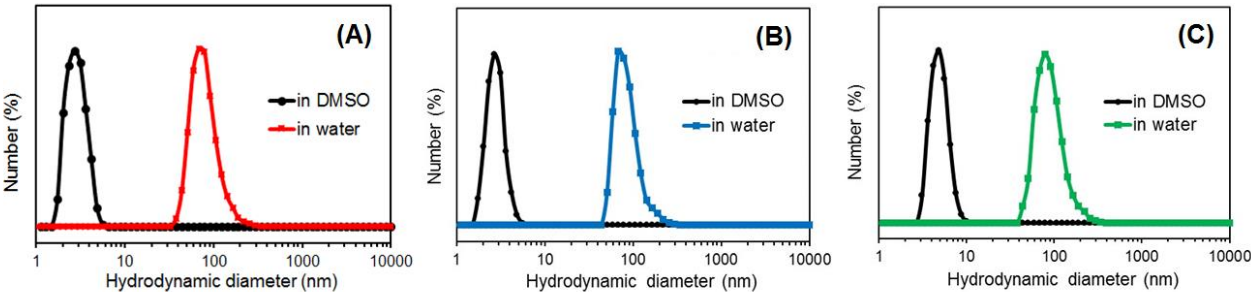

Figure S2. DLS size distributions of (A) **SCP1**, (B) **SCP2**, and (C) **SCP3** copolymers in DMSO and aqueous medium at 25 ^{o}C (concentration: 1 mg/mL).

Self-assembly study by ^{1}H NMR spectroscopy. Since DLS measurements suggested formation of particles (self-assembly of copolymers) in aqueous medium, we recorded ^{1}H NMR spectra of copolymers in D_2O and DMSO-d_6 solvents. Since DMSO-d_6 is good solvent for both the lipophilic and hydrophilic segments present in the synthesized copolymers, all the peaks corresponding to the different protons in the copolymer appear in the ^{1}H NMR spectrum (Figure S3). However, cholic acid segments are insoluble in D_2O. So, the peak at 0.60 ppm, responsible for methyl group of cholate moiety (marked), is absent in the ^{1}H NMR spectrum of **SCP2** in D_2O (Figure S3). Similar ^{1}H NMR spectra were obtained for **SCP1** and **SCP3** in DMSO-d_6 and D_2O (data not show). These results indicate formation of self-assembled higher-order structure by the copolymers in water, where the MAECA moiety forms the core as the protons from this unit did

not appear in the spectra because of suppressed molecular motion of the aggregated cholate moieties.[2]

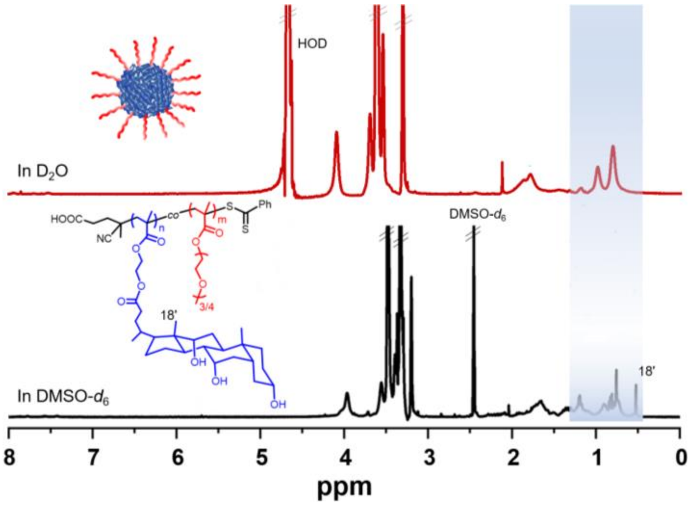

Figure S3. ^{1}H NMR spectra of **SCP2** in DMSO-d_6 and D_2O at 25 ^oC.

Determination of critical aggregation concentration (CAC) by fluorescence spectroscopy. We further determined CAC *via* fluorescence spectroscopy using pyrene as fluorescence hydrophobic probe.[3] A pyrene stock solution of 60 μL in acetone was added into different glass vials having different polymer concentrations and overall volume was kept to 3 mL (by adding water). Thus, a series of copolymer solutions were obtained (from 1 mg/mL to 0.25 mg/L) with constant pyrene concentration (1.0×10^{-7} M) in each vial. Each of the vials were sonicated for 10 min and kept for 4 h under open condition at room temperature to evaporate acetone completely. The pyrene emission intensity ratios I_{384}/I_{372} with excitation at 337 nm,[4] were recorded to determine CAC of the copolymers through linear fitting of the data against logarithmic concentrations and extrapolating the fitted lines on the curve. One representative plot of emission spectra of **SCP2** in water is shown below (Figure S4A). The fluorescent intensity

ratio I_{384}/I_{372} against logarithmic concentration of **SCP2** copolymer is also drawn, see Figure S4B. CAC of **SCP1**, **SCP2** and **SCP3** copolymers were found to be 6.9, 6.4 and 5.3 μg/mL respectively. This result indicates that our copolymers remain in aggregated state at the concentration used for the investigation of their ionophoric activities.

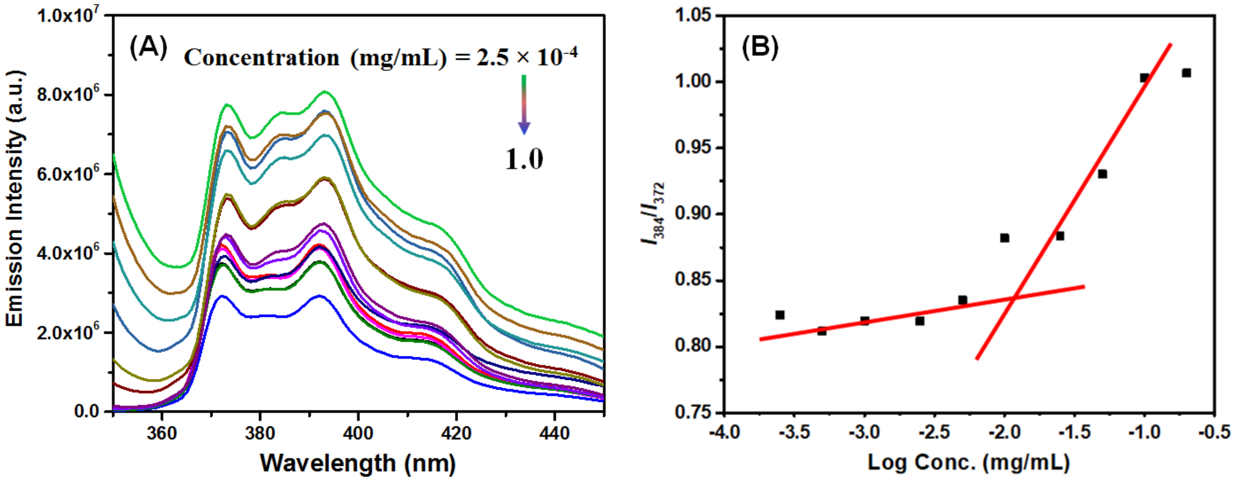

Figure S4. (A) Emission spectra of pyrene at various concentrations of **SCP2** in water, and (B) plot of intensity ratio I_{384}/I_{372} versus logarithmic concentrations of **SCP2**.

Morphological evaluation by TEM. The copolymer morphologies were also recorded in dry state from their aqueous solutions. The microscopic data revealed that all the copolymers exhibit spherical micellar morphology, as shown in Figure S5. **SCP1** showed an average size of 64 nm (Figure S5A), whereas we observed an average size of 67 nm for the **SCP2** (Figure S5B).

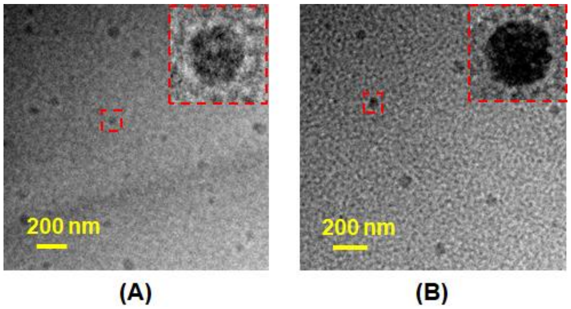

Figure S5. TEM images of **SCP1** (A) and **SCP2** (B). Inset pictures show enlarged view of the red marked squares.

Ionophoric activity

General procedures. Egg yolk phosphatidylcholine (EYPC), 8-hydroxypyrene-1,3,6-trisulfonic acid trisodium salt (HPTS) and Calcein were from Sigma; Triton® X-100 and HEPES buffer were from Fluka; all salts were of the best grade available from Aldrich and were used without further purification. Liposomes were prepared by extrusion using a 10 mL LipexTM Thermobarrel EXTRUDER (Northern Lipids Inc.) connected to a thermostatic bath maintained at 25 °C. 100 nm polycarbonate membranes were Nucleopore Track-Etch Membranes from Whatman. Fluorescence spectra were recorded on a Varian Cary Eclipse fluorescence spectrophotometer. All fluorimetric measurements were performed at 25 °C. The ionophore concentration is given in percent with respect to the total concentration of lipids. Mother solutions of polymers were prepared in water.

HPTS assay. In this assay HPTS, a fluorescent pH indicator with a pKa of 7.2, is trapped in the inner water pool of large unilamellar vesicles (LUVs, 100 nm diameter) made by egg yolk phosphatidylcholine (EYPC). The lipid suspension is prepared in water buffered at pH 7 and

containing 100 mM NaCl. The ionophore is added and after 50 seconds a base pulse is applied by addition of NaOH to generate a 0.6 units pH gradient between the bulk water and the liposome inner water pool. The increase of the HPTS fluorescence emission in response to the applied transmembrane pH-gradient indicates basification of the inner water pool which may be derived either from H^+ efflux or OH^- influx, balanced by the opposite transport of ions of the same charge or by the symport of ions of the opposite charge. After 350 seconds the vesicles are lysed by addition of a surfactant (Triton X-100) allowing to measure the maximal intensity of fluorescence which is then used to normalize the data (Figure S6).

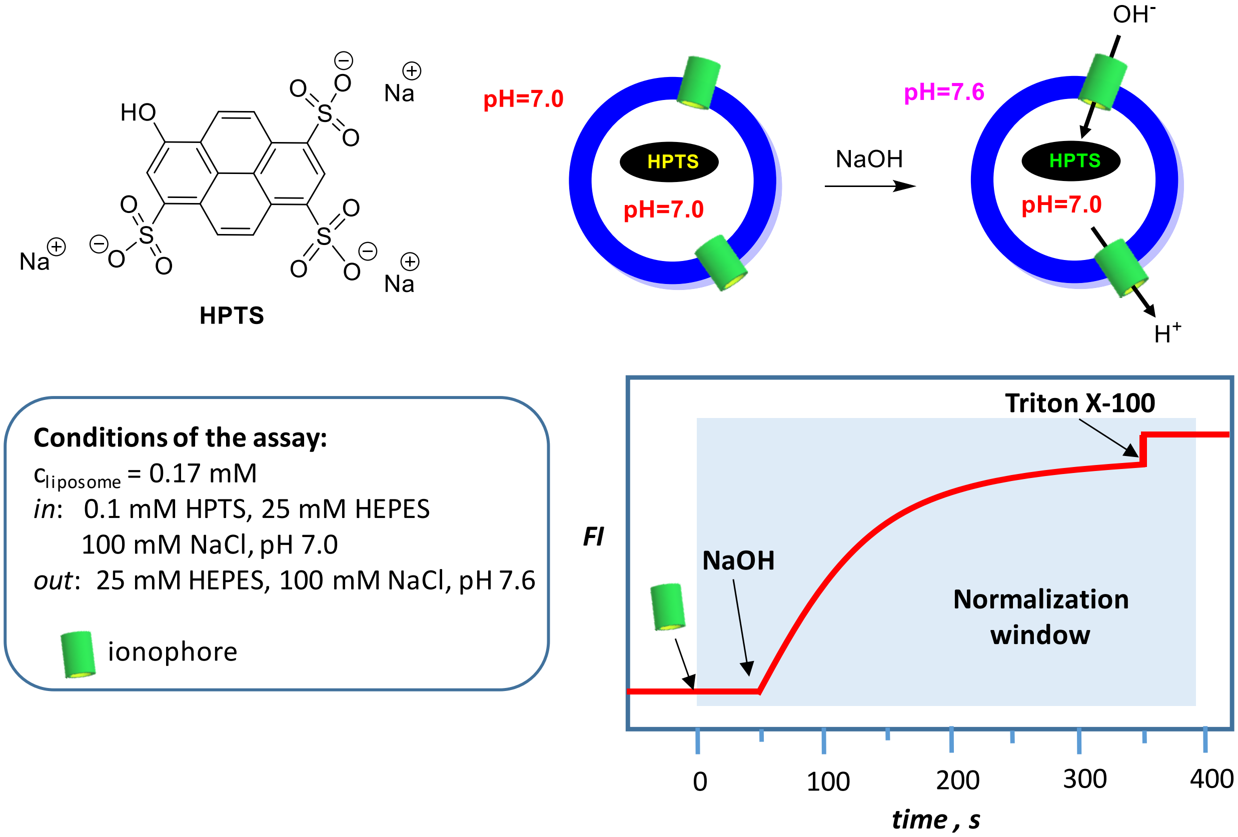

Figure S6. Structure of HPTS and schematic illustration of the HPTS assay (top) and fluorescence kinetics experiment of ion transport (bottom).

Procedure for the HPTS assay. 15 mg of EYPC (20 µmol) was first dissolved in chloroform and evaporated under Ar-flux to form a thin film and then dried under high vacuum for 3 h. The lipid cake was hydrated in 1.5 mL of 0.1 mM HPTS solution (HEPES 25 mM, 100 mM NaCl, pH 7.0) for 30 min at 40°C. The lipid suspension was submitted to 5 freeze-thaw cycles (-196°C/40°C) using liquid nitrogen and a thermostatic bath, and then extruded under nitrogen pressure (15 bar) at room temperature (10 extrusions through a 0.1 µm polycarbonate membrane). The LUV suspension was separated from extravesicular dye by size exclusion chromatography (SEC) (stationary phase: pre-packed column Sephadex™ G-25, mobile phase: HEPES buffer 25 mM, 100 mM NaCl, pH 7.0) and diluted with HEPES buffer (HEPES 25 mM, 100 mM NaCl, pH 7.0) to give a stock solution with a lipid concentration of 5 mM (assuming 100% of lipids were incorporated into liposomes). 104 µL of the lipid suspension were placed in a fluorimetric cell and diluted to 3040 µL with HEPES buffer (HEPES 25 mM, 100 mM NaCl, pH 7.0). The total lipid concentration in the fluorimetric cell was 0.17 mM. An aliquot of solution of the ionophore in water (10-70 µL of the appropriate mother solution in order to obtain the desired molcompound/mollipid ratio) was then added to the lipid suspension and the cell was incubated at 25°C for 5 min. After incubation, the time course of fluorescence was recorded for 50 s monitoring the HPTS emission at 510 nm with excitation wavelengths set alternatively at 403 and 460 nm on a 0.5+0.5 s cycle. Then 50 µL of 0.5 M NaOH were rapidly added through an injector port and the fluorescence emission was recorded for 350 s. In each experiment, maximal changes in dye emission were obtained by final lysis of the liposomes with a detergent (40 µL of 5% aqueous Triton® X-100). The data set consists of emission intensities at 510 nm modulated by alternating excitation at 403 nm and 460 nm on a 0.5+0.5 s cycle. The concentration of the conjugate base form of HPTS is related to the emission intensity at 510 nm

during the period in which the dye is excited at 460 nm (E_{460}) while the concentration of the protonated form is related to the emission intensity at 510 nm during the period in which the dye is excited at 403 nm (E_{403}). Fluorescence time courses were normalized using the following equation, where the subscripts 0, ∞ and t denote the emission ratio before the base pulse, after detergent lysis, and at an intermediate time, respectively. Normalization of the data is performed for an easier comparison between different batches of liposome preparation which may slightly differ for dye content.

$$Normalized\ FI = \frac{\left(\frac{E_{403}}{E_{460}}\right)_t - \left(\frac{E_{403}}{E_{460}}\right)_0}{\left(\frac{E_{403}}{E_{460}}\right)_\infty - \left(\frac{E_{403}}{E_{460}}\right)_0} * 100$$

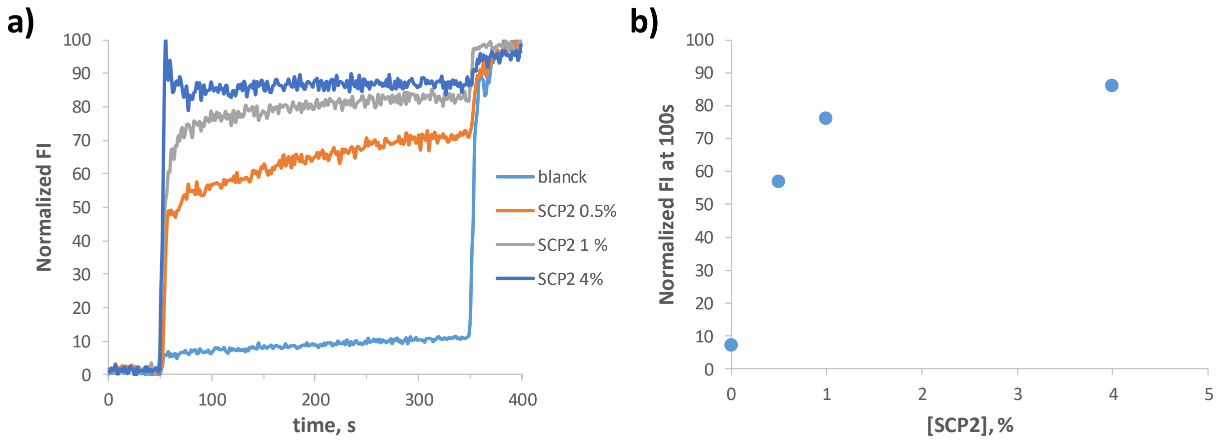

Figure S7. a) Normalized fluorescence change in HPTS fluorescence emission (FI) as a function of time in the presence of different concentrations of **SCP2**. b) Normalized FI measured at 100 s as a function of **SCP2** concentration. The concentrations of **SCP2** are reported in the Figures and are given in mol% with respect to the concentration of lipids.

Calcein leakage assay. In this assay calcein is trapped in the inner water pool of large unilamellar vesicles (LUVs, 100 nm diameter) made by egg yolk phosphatidylcholine (EYPC). Calcein is a self-quenching fluorescent dye which is not membrane permeable. When trapped in the liposomes at high concentration (50 mM) its fluorescent emission is almost completely quenched due to the self-quenching process. However, if a ionophore forms pores large enough to allow the dye to leak from the liposome and to dilute in the bulk water the self-quenching is removed and the fluorescence emission intensity increases substantially. This is schematically illustrated in Figure S8 where the ionophore is added at 50 s. Then after 450 seconds the vesicles are lysed by addition of a surfactant (Triton X-100) allowing to measure the maximal intensity of fluorescence which is then used to normalize the data.

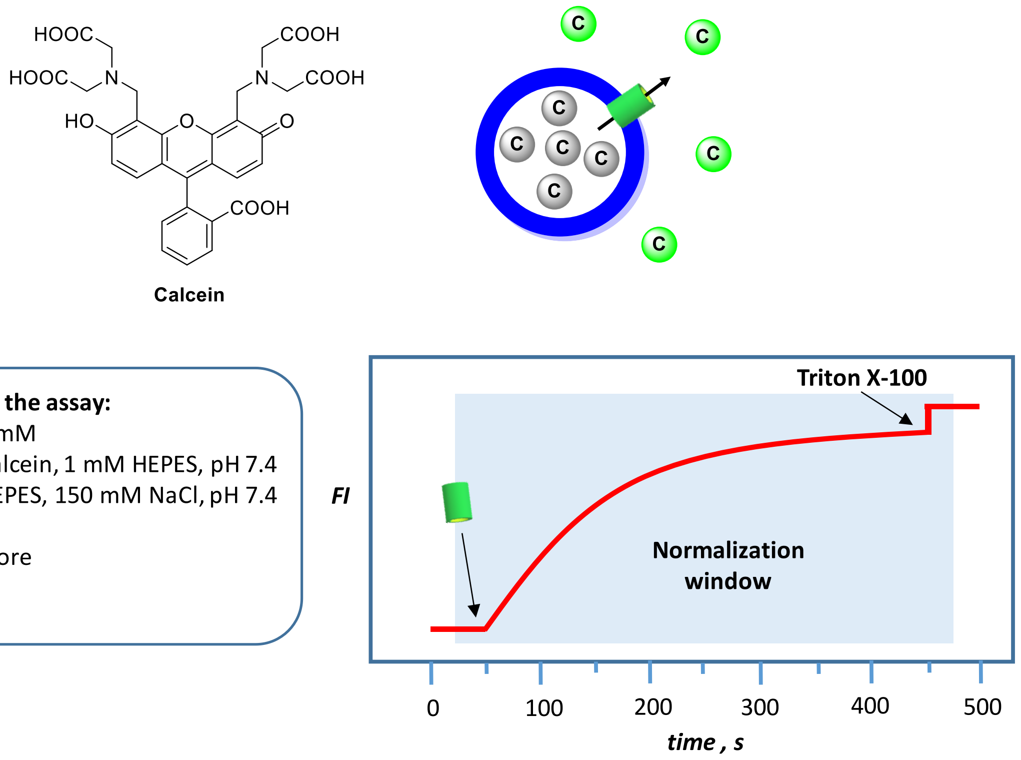

Figure S8. Structure of calcein and schematic illustration of the leakage assay (top) and fluorescence kinetics experiment calcein leakage (bottom).

Procedure for the Calcein leakage assay. 15 mg of EYPC (20 µmol) was first dissolved in chloroform and evaporated under Ar-flux to form a thin film and then dried under high vacuum for 3 h. The lipid cake was hydrated in 2.0 mL of 50 mM calcein solution (1 mM HEPES, pH 7.4) for 30 min at 40°C. The lipid suspension was submitted to 5 freeze-thaw cycles (-196°C/40°C) using liquid nitrogen and a thermostatic bath, and then extruded under nitrogen pressure (15 bar) at room temperature (10 extrusions through a 0.1 µm polycarbonate membrane). The LUV suspension was separated from extravesicular dye by size exclusion chromatography (SEC) (stationary phase: Sephadex™ G-75, mobile phase: HEPES buffer 1 mM, 150 mM NaCl, pH 7.4, 1 x 25 cm column). The LUV suspension was placed in a fluorimetric cell and diluted with the same buffer in order to achieved 0.1 mM concentration of lipid (assuming 100% of lipid was incorporated into liposomes). Calcein emission was monitored at 520 nm with excitation at 490 nm. An aliquot of solution of the ionophore (2-70 µL of the stock solution in water in order to obtain the desired $mol_{compound}/mol_{lipid}$ ratio) was then added to the lipid suspension and the time course of fluorescence was recorded was recorded for 500 s at 25 °C. Maximal changes in dye emission were obtained by lysis of the liposomes with detergent (40 µL of 5% aqueous Triton X-100). The fluorescence data were then normalized using the following equation where the subscript 0, ∞ and t denote the emission intensity before the addition of the ionophore, after detergent lysis, and at some intermediate time respectively. Normalization of the data is performed for an easier comparison between different batches of liposome preparation which may slightly differ for dye content.

$$Normalized\ FI = \frac{F_t - F_0}{F_\infty - F_0} * 100$$

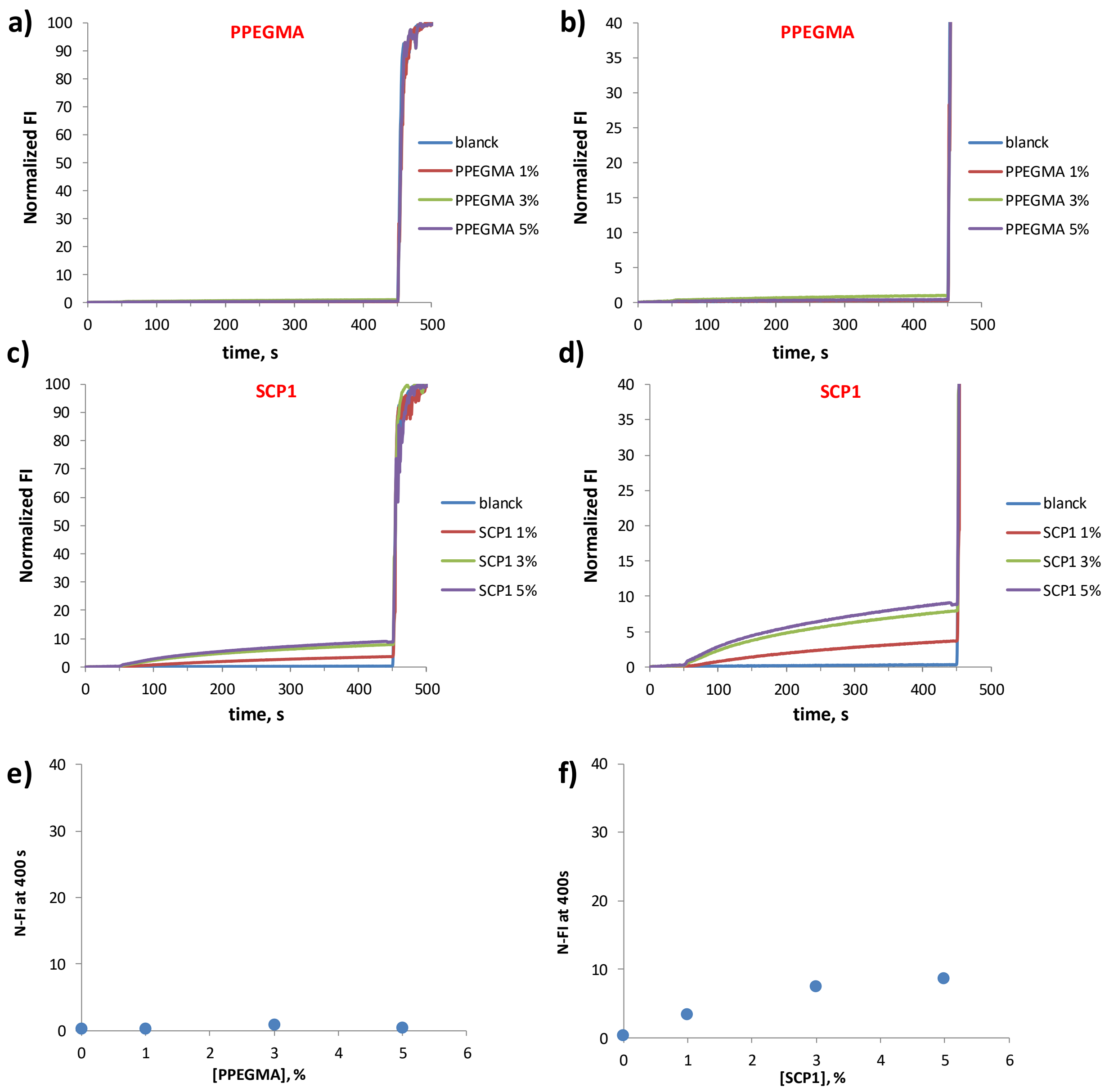

Figure S9. Time course of calcein leakage from EYPC LUVs (100 nm diameter, 50 mM calcein, 1mM HEPES, pH 7.4, 0.1 mM lipids concentration) in the presence of increasing concentration of **PPEGMA** (a and b, b is the expansion of plot a) and **SCP1** (c and d, d is the expansion of plot c). The polymer was added at 50 s and the liposomes were lysed with Triton X-100 (40 µl, 5% water solution) at 450 s. Percent leakage of calcein after 400 s as a function of **PPEGMA** (e) and **SCP1** (f) concentration. The concentrations of polymers are reported in the Figures and are given in mol% with respect to the concentration of lipids.

a)

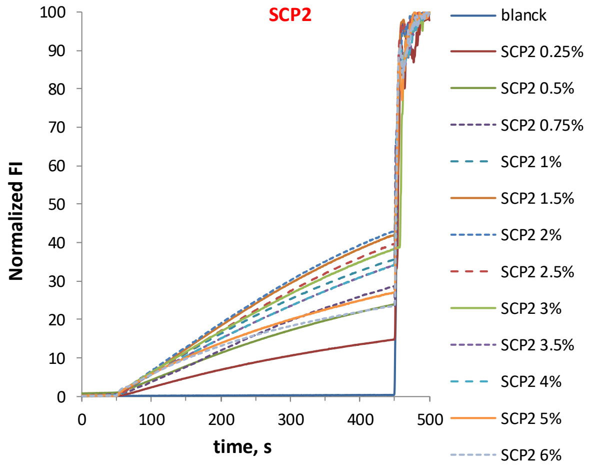

b)

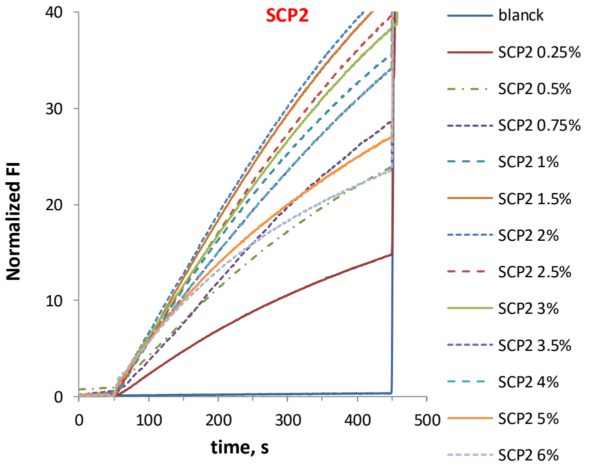

c)

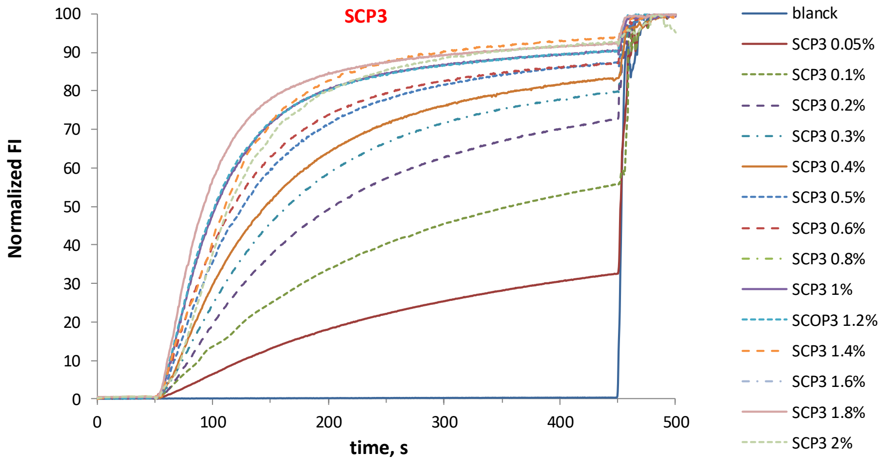

Figure S10. Time course of calcein leakage from EYPC LUVs (100 nm diameter, 50 mM calcein, 1mM HEPES, pH 7.4, 0.1 mM lipids concentration) in the presence of increasing concentration of **SCP2** (a and b, b is the expansion of plot a) and **SCP3** (c). The polymer was added at 50 s and the liposomes were lysed with Triton X-100 (40 μl, 5% water solution) at 450 s. The concentrations of polymers are reported in the Figures and are given in mol% with respect to the concentration of lipids.

References

(1) Bauri, K.; Pant, S.; Roy, S. G.; De, P. Dual pH and Temperature Responsive Helical Copolymer Libraries with Pendant Chiral Leucine Moieties. *Polym. Chem.* **2013**, *4*, 4052-4060.

(2) Pal, S.; Roy, S. G.; De, P. Synthesis via RAFT Polymerization of Thermo- and pH-Responsive Random Copolymers Containing Cholic Acid Moieties and Their Self-Assembly in Water. *Polym. Chem.* **2014**, *5*, 1275-1284.

(3) Goswami, K. G.; Mete, S.; Choudhury, S. S.; Sar, P.; Ksendzov, E.; Mukhopadhyay, C. D.; Kostjuk, S. V.; De, P. Self-Assembly of Amphiphilic Copolymers with Sequence-Controlled Alternating Hydrophilic–Hydrophobic Pendant Side Chains. *ACS Appl. Polym. Mater.* **2020**, *2*, 2035-2045.

(4) Maiti, B.; Maiti, S.; De, P. Self-Assembly of Well-Defined Fatty Acid Based Amphiphilic Thermoresponsive Random Copolymers. *RSC Adv.* **2016**, *6*, 19322-19330.
